# Protein Binding During Mouse Skin Carcinogenesis by 9,10-Dimethyl-1,2-Benzanthracene. The Effect of Copper Acetate and the Non-random Distribution of Induction Times Among Mice Given Identical Treatment

**DOI:** 10.1038/bjc.1964.89

**Published:** 1964-12

**Authors:** G. Fare


### PROTEIN BINDING DURING MOUSE SKIN CARCINOGENESIS BY 9,10-DIMETHYL-1,2-BENZANTHRACENE. THE EFFECT OF COPPER ACETATE AND THE NON-RANDOM DISTRIBUTION OF INDUCTION TIMES AMONG MICE GIVEN IDENTICAL TREATMENT

G. FARE

*From the Cancer Research Laboratories, Department of Pathology, Medical School, Birmingham* 15

Received for publication September 15, 1964

DIETARY copper acetate affords a good degree of protection against liver damage in the rat induced by 4-dimethylaminoazobenzene (Howell, 1958), thioacetamide (Fare, 1965) and 3-methoxy-aminoazobenzene and its N-methyl derivative (Fare and Howell, 1964). No protection was given against the induction of skin and ear duct tumours by the 3-methoxy dyes. It has been suggested (Fare, 1964) that copper may act by binding to liver protein in competition with carcinogen. Since there is evidence that the carcinogenic hydrocarbons bind to skin components, notably proteins, and since it has been suggested that such binding may be an essential factor in carcinogenesis, it was thought that painting with a carcinogen solution also containing copper might give some protection.

The powerful carcinogen 9,10-dimethyl-1,2-benzanthracene (DMBA, new nomenclature 7,12-dimethyl-benz-(a)-anthracene) was used in acetone and in olive oil with and without copper acetate in each case. The tumour incidence was recorded, and protein bound copper and hydrocarbon in the skin were determined in the early stages.

### MATERIALS AND METHODS

*Mice.*—290 White male mice were randomised and housed in boxes of 5. Water and a vitamin-supplemented pellet food were available *ad libitum.*

*Chemicals.*—DMBA (Eastman Kodak) and cupric oxyacetate hexahydrate (CuAc, Hopkin and Williams) were used without purification. Analar acetone was redistilled before use and olive oil was used as supplied.

Four solutions were prepared at weekly intervals and stored at room temperature.

0·05 per cent DMBA in acetone

0·05 per cent DMBA + 0·15 per cent CuAc in acetone

0·05 per cent DMBA in olive oil

0·05 per cent DMBA + 0·15 per cent CuAc in olive oil

The first three solutions were prepared by shaking in the cold. The fourth was made up by adding an appropriate volume of a solution of the copper salt in acetone to 0·05 per cent DMBA in olive oil and removing the acetone at room temperature under vacuum. The resulting mixture was stable for the one week period. It is not known whether the copper was in chemical combination with the oil.

*Plan of experiment*

(i) *Incidence of tumours.*—4 × 40 Mice were painted twice weekly between the scapulae with 0·2 ml. of the above solutions and the tumour yield was noted in the middle of each week. Hair was shaved from the target area at the beginning of the experiment and at intervals where required with the olive oil paintings. Application of DMBA in acetone with or without CuAc prevented hair growth.

When tumours developed, the volume of solution applied was decreased so that only those parts within the painted area without tumours were treated. When multiple tumours had been produced, painting was discontinued. Records were kept of each mouse so that tumours which regressed or coalesced could be noted. The experiments were terminated when it became apparent that a large number of the animals would shortly have to be killed for humanitarian reasons.

(ii) *Protein binding.*—4 Groups of 30 mice were painted as before. Great care was taken to paint at an exact time each week. 1 Box of 5 mice was painted with acetone alone and one box with olive oil alone as controls. Exactly 24 hours after the 2nd, 4th, 6th, 8th, 10th and 12th paintings, i.e. after treatment for 1–6 weeks inclusive, a box of 5 mice was killed from each experimental group (the controls were killed after 6 weeks), and the skin from the treated areas removed. The combined skins from each group were chopped up finely with scissors and extracted in a mechanical blendor with 10 per cent TCA in 85 per cent ethanol. The homogenate was boiled, centrifuged and washed with volumes of hot extraction mixture.

The tissue was then treated in a Soxhlet apparatus as follows, using redistilled solvents throughout.

Ethanol for 8 hours—dehydrates and extracts some fat and carcinogen.

Acetone for 4 hours then chloroform for 8 hours—completes defatting.

Benzene for 8 hours—removes hydrocarbons resistant to ethanol extraction.

Ethanol for 4 hours—removes still more fluorescent material.

After the above exhaustive extraction scheme, further solvent treatment removed no further hydrocarbon or lipid and the tissue was considered to be essentially protein.

The skin protein was stored in a vacuum desiccator for 48 hours to remove traces of solvent, then in a desiccator at atmospheric pressure over phosphorus pentoxide to remove traces of water. The tissue was then allowed to equilibrate in the atmosphere on an analytical balance until constant weight was attained. The tissue was then stored in sealed vials until required.

Protein content was assessed by Kjeldahl nitrogen assay. Bound copper was determined by a colorimetric method using biscyclohexanone oxalyldihydrazone (Nilsson, 1950) and bound hydrocarbon as follows:

A sample of tissue (200 mg.) was refluxed on a water bath for 6 hours with 4N KOH in 85 per cent redistilled ethanol. The extract was diluted with water and hydrocarbons were extracted with repeated 10 ml. volumes of redistilled Analar benzene. The aqueous residue was made just acid with HCl and again extracted exhaustively with benzene.

The fluorescences of both solutions were measured in an EIL direct reading fluorimeter model 27A (incident light 3655 Å Hg line, good transmission from 4800–6100 Å).

Fluorescence was expressed in terms of a standard amount of DMBA which had been mixed with 200 mg. of normal mouse skin protein and subjected to the hydrolysis and extraction procedure. Results are expressed as $\mu g$. DMBA although the hydrocarbons liberated by hydrolysis would be of diverse identity.

FIG. 1.—Tumour incidence in mice painted twice weekly with 0·2 ml. of 0·05 per cent DMBA (○) or with 0·2 ml. of 0·15 per cent CuAc + 0·05 per cent DMBA (●) in olive oil. Thirty-eight mice at risk in each group.

### RESULTS

*Incidence of tumours.*—With olive oil as the solvent, there was no detectable difference in incidence of skin tumours between the DMBA and the CuAc+DMBA solutions (Fig. 1) nor in the proportion of these which were considered to be malignant by macroscopic or microscopic examination (about 2 per cent).

Two mice died in each group before the first tumour was found after 11·5 weeks treatment. The remainder all survived until the end of the 24th week when the experiment was ended, with about 80 per cent of the animals with tumours.

The mice painted with copper developed a greater number of tumours per tumour-bearing mouse (7·50) after 24 weeks than did mice painted with DMBA alone (5·11). The total yield of tumours is given on Fig. 2.

The copper salt in the acetone medium had a marked effect on tumour induction. With both solutions, the first tumour was found after 6·5 weeks, i.e. about one half the induction time in olive oil. During this time, the acetone solutions caused dryness and excoriation of the skin whereas with olive oil as the solvent no changes were observed until tumours appeared Thirty-nine mice were at risk in either group, all of which survived until the experiment was terminated after 17 weeks. Mice painted with CuAc + DMBA showed a 100 per cent incidence after only 14·5 weeks at which time the mice treated with DMBA alone had developed tumours in about 55 per cent yield (Fig. 3). Fig. 4 shows

FIG. 2.—Total number of tumours produced in the mice painted with DMBA (○) or CuAc +DMBA (●) in olive oil. Thirty-eight mice in each group throughout.

the total number of tumours produced in each group. In contrast with the olive oil experiments, both groups showed identical numbers of tumours per tumour-bearing mouse (alone 5·83, with copper 5·95). Again, about 2 per cent of all tumours were considered to be malignant.

Although the induction time varied among mice given identical treatment, it was noticed that the mice in any one box often had similar induction times. Table I gives an instance of this. It will be noticed that all mice in box B developed tumours during the 17th week, 4 mice in box E all did so during the

TABLE I.—*Times in Weeks at which Skin Tumours were First Detected in 7 Boxes of 5 Mice Treated with Both Chemicals in Olive Oil*

| Mouse number | Box A | Box B | Box C | Box D | Box E | Box F | Box G |
|---|---|---|---|---|---|---|---|
| 1 | 13·5 | 16·5 | 17·5 | — | 19·5 | 19·5 | 18·5 |
| 2 | 13·5 | 16·5 | 17·5 | — | 21·5 | 19·5 | 18·5 |
| 3 | 15·5 | 16·5 | 18·5 | — | 21·5 | 21·5 | 18·5 |
| 4 | 17·5 | 16·5 | 18·5 | — | 21·5 | 24·5 | 19·5 |
| 5 | 20·5 | 16·5 | — | — | 21·5 | — | 19·5 |

The mice in each box are numbered arbitrarily from 1 to 5 in the order in which they developed tumours. The box identifications of A–G are also arbitrary. The eighth box in this group is not included since only three of the five mice survived to be " at risk ".

FIG. 3.—Tumour incidence in mice painted twice weekly with 0·2 ml. of 0·05 per cent DMBA (○) or with 0·2 ml. of 0·15 per cent CuAc + 0·05 per cent DMBA (●) in acetone. Thirty-nine mice at risk in each group.

FIG. 4.—Total number of tumours produced in the mice painted with DMBA (○) or CuAc +DMBA (●) in acetone. Thirty-nine mice in each group at risk throughout.

22nd week and 3 mice in box G also developed tumours simultaneously. Although 4 boxes of 5 mice produced tumours, no mouse in box D did.

This phenomenon was also exhibited in the other three groups, and the detailed protocols were submitted to statistical analysis. There were 7 boxes with a full complement of 5 mice at the end of each experiment. Boxes where one or more mice had died were excluded from the analysis. In both groups where two mice died, they were 2 mice from the same box.

Analysis of variance showed that there was a difference in the times at which tumours arose in the various boxes receiving identical treatment significant at the 0·1 per cent level.

FIG. 5.—Copper bound to skin protein in mice painted with 0·05 per cent DMBA + 0·15 per cent CuAc in olive oil (top) or in acetone (below).

*Protein binding experiment.*—None of the 130 animals developed tumours during the 6 weeks duration of this experiment but 6 mice died. There were no differences between the 4 groups in nitrogen content of the skin protein samples. These ranged from 13·2 to 14·7 per cent, mean 14·1.

The copper content of the normal skin was 2·32 (olive oil) and 2·49 (acetone) μg. Cu/g. protein nitrogen. The application of DMBA alone in either solvent gave levels similar to this, from 2·26 to 2·58, mean 2·48. When copper acetate was included, there was some binding of copper to skin protein (Fig. 5) with about 25 per cent more copper being bound to protein from olive oil than from acetone.

Protein-bound hydrocarbon is given in Fig. 6. In all four groups the total recovered fluorescence rose to a maximum value after between the eighth and tenth application which was subsequently maintained. Presumably after each individual application, the bound hydrocarbon reaches a maximum value and

FIG. 6.—Hydrocarbons bound to skin protein in mice painted twice weekly with 0·2 ml. of DMBA in acetone (A) or olive oil (B) and with 0·2 ml. of 0·15 per cent CuAc + 0·05 per cent DMBA in acetone (C) or olive oil (D). The total column height is the total amount of fluorescent material extracted, expressed as DMBA. The height of the cross-hatched column represents the amount found in the alkaline hydrolysate. The difference is the value found in the acidified extract.

then diminishes, but the cumulative effect is to produce a "saturation" level. The proportion of the fluorescence recovered from the alkaline and acidified extracts varied ; generally the former accounted for the greater part of the hydrocarbon.

With either solvent, there was rather less binding of hydrocarbon when copper also was applied. As with copper binding, there was more binding of DMBA

from olive oil than from acetone solution, irrespective of whether copper was included in the painting solution.

### DISCUSSION

The fact that DMBA alone in the two different vehicles gives rise to tumours at different rates is not surprising in view of the different characteristics of these solvents. Acetone is so volatile that a proportion of the carcinogen remains on the skin from which it may easily be removed by friction. Acetone is a good solvent for fat, so that the DMBA which is carried into the skin is likely to come into contact with lipid. Olive oil will not evaporate, and a higher dose of the carcinogen is likely to be deposited within the tissue. However, the olive oil solution is likely to be more palatable than acetone and a considerable loss by licking is likely. The oil may not penetrate the skin as easily as acetone, but because of its immiscibility with water, it is likely to persist for some time in the tissue.

It is surprising, however, that the inclusion of copper has an effect only in the one solvent. There is binding to skin protein in each case, indeed slightly more so from olive oil, the solvent where there was no effect. The ultra-violet absorption spectrum of DMBA was unchanged after one week's standing of the DMBA + CuAc solution in acetone as regards both peak wavelengths and peak heights. It can therefore be assumed that the presence of copper had no effect on the carcinogen *in vitro.*

With both solvents, rather less hydrocarbon was bound in the presence of copper. From this it could be inferred that the DMBA and metal were binding to similar proteins. Heidelberger and Moldenhauer (1956) used maleic anhydride and other chemicals to lessen the incidence of papillomas by hydrocarbons and they obtained reduced protein binding, especially to water-soluble proteins. In this case, less hydrocarbon binding is associated with increased yields of papillomas (acetone) or the same yields (olive oil).

Although DMBA was bound to a greater extent from olive oil solution regardless of the fact that the acetone solution gave much the shorter induction time, this need not be taken as evidence that binding to skin protein is irrelevant to experimental skin carcinogenesis. Such binding to protein is likely to be quite general and in theory only a few or indeed one key protein need be involved for fundamental changes to occur. However, it is of course possible that binding to other cellular constituents (nucleic acids or nucleotides for example) is the essential process and that protein binding is just incidental.

Be that as it may, copper binds to liver protein in circumstances where protection is given against tumour formation (Fare, 1964) and binds to skin protein in circumstances where the same or increased yields of tumours are obtained.

### *The non-random distribution of tumour induction time*

A most interesting result to emerge from these experiments is the realisation that among mice given identical treatment, there is a significant difference between boxes as regards the times at which the mice contained therein first develop tumours. The experiments were carried out at a time when our mouse colony was being re-equipped with plastic boxes, and the mice in these experiments were housed in brand-new boxes. Contamination with other chemicals, including carcinogens, from previous experiments was therefore excluded. Spouts of water bottles were examined routinely before the start of the experiment under a UV lamp and were found to be carcinogen free. The mice were first randomised in a two stage process, first by human selection from a pool and then by blind selection of numbers using a mechanical device. They received identical food, were painted by the same operator (GF) throughout using the same pipette and solution for all the mice in each group on any particular day and were painted in a different order each time so that operator fatigue would not be a factor.

The implications, if these results are confirmed in subsequent experiments, appear to be of some moment. Since tumour development time appears to be affected by which particular box a mouse finds itself in, presumably the results of experiments involving the application of carcinogens to the skin would differ if the mice were housed in boxes of, for example, 10 rather than our customary five. An experiment is in progress in which equal numbers of mice are housed in boxes of 5, boxes of 3 and singly. Identical treatment is being given to all animals taking all available precautions. If it is found that the tumour induction time for the three separate groups varies, then in future, results of tumour incidences must always be compared only between experiments in which the boxes held an arbitrary number of mice.

### SUMMARY

1. Mice painted twice weekly with 0·2 ml. of 0·05 per cent DMBA in acetone show a shorter tumour induction time compared with mice treated similarly with olive oil as the vehicle.

2. Addition of 0·15 per cent cupric oxyacetate to the carcinogen solution gives an accelerated tumour yield in acetone solution but is without effect when olive oil is the solvent.

3. There is less binding of carcinogen to skin protein, measured fluorimetrically, when copper acetate is included in either solution. The metal binds to a greater extent from the olive oil solution than from acetone.

4. With DMBA alone, there is rather more binding from the oil than from acetone.

5. There is a tendency within each treated group for mice in the same boxes to have similar tumour induction times. The times at which tumours first arise in boxes in any one group varies significantly from box to box.

I am grateful to Dr. D. L. Woodhouse for his interest and advice. I am also indebted to Dr. Waterhouse (Department of Medical Statistics, Queen Elizabeth Hospital, Birmingham 15) for the statistical analyses.

This work was carried out partly under the tenure of a Medical Research Council scholarship and was supported by the Birmingham branch of the British Empire Cancer Campaign for Research.

### REFERENCES

FARE, G.—(1964) *Biochem. J.*, **91**, 473.—(1965) *Amer. J. Path.*, in press.

*Idem* AND HOWELL, J. S.—(1964) *Cancer Res.*, **24**, 1279.

HEIDELBERGER, C. AND MOLDENHAUER, M. G.—(1956) *Ibid.*, **16**, 442.

HOWELL, J. S.—(1958) *Brit. J. Cancer*, **12**, 594.

NILSSON, G.—(1950) *Acta chem. scand.*, **4**, 205.


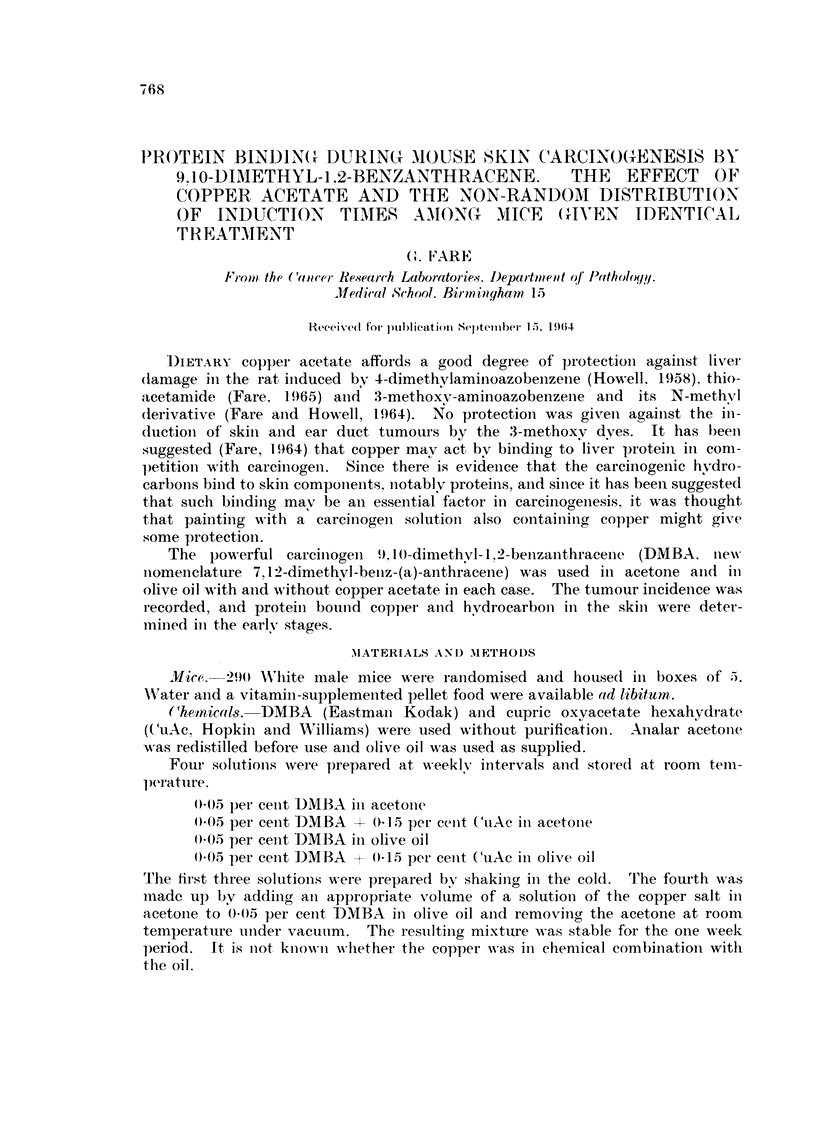

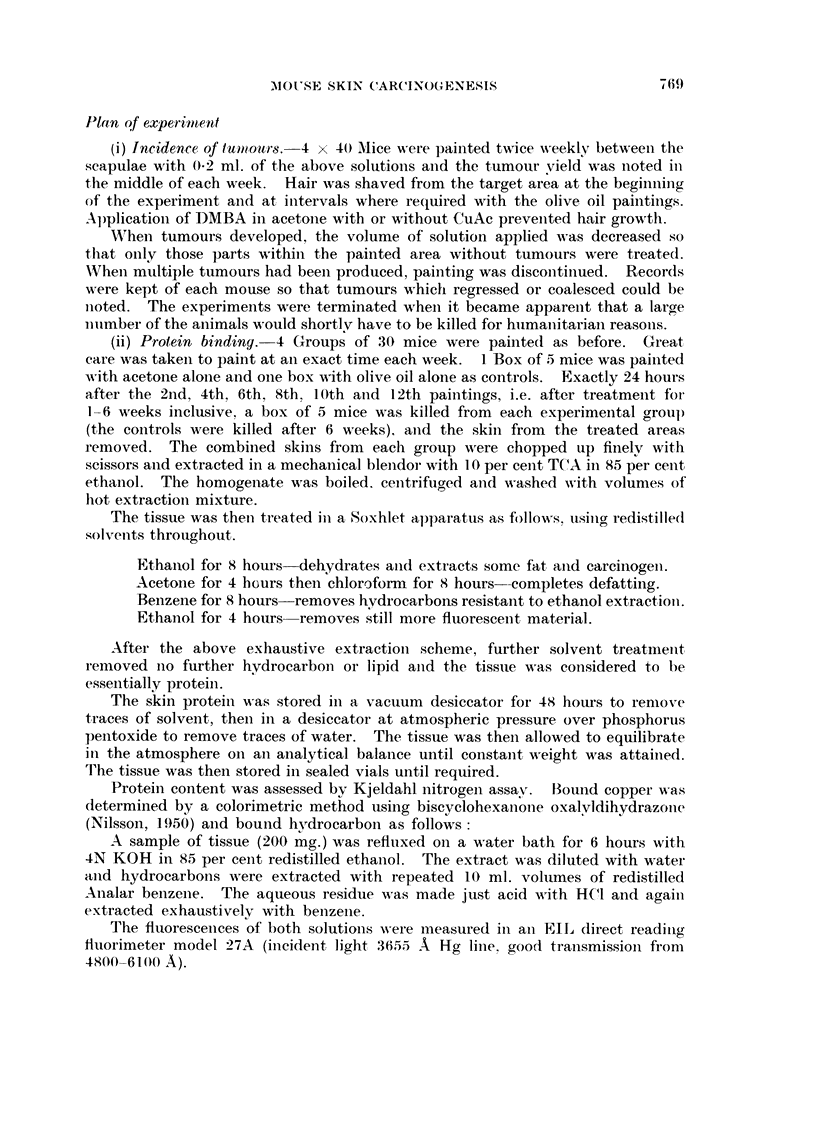

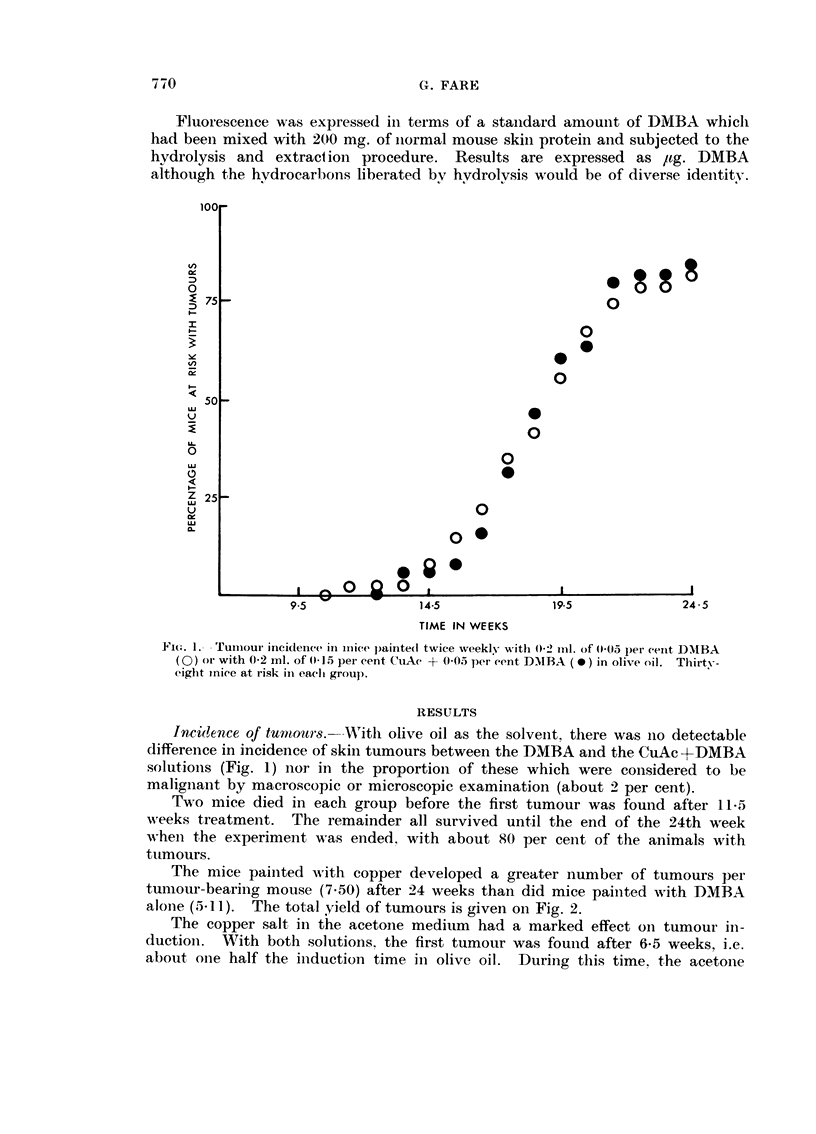

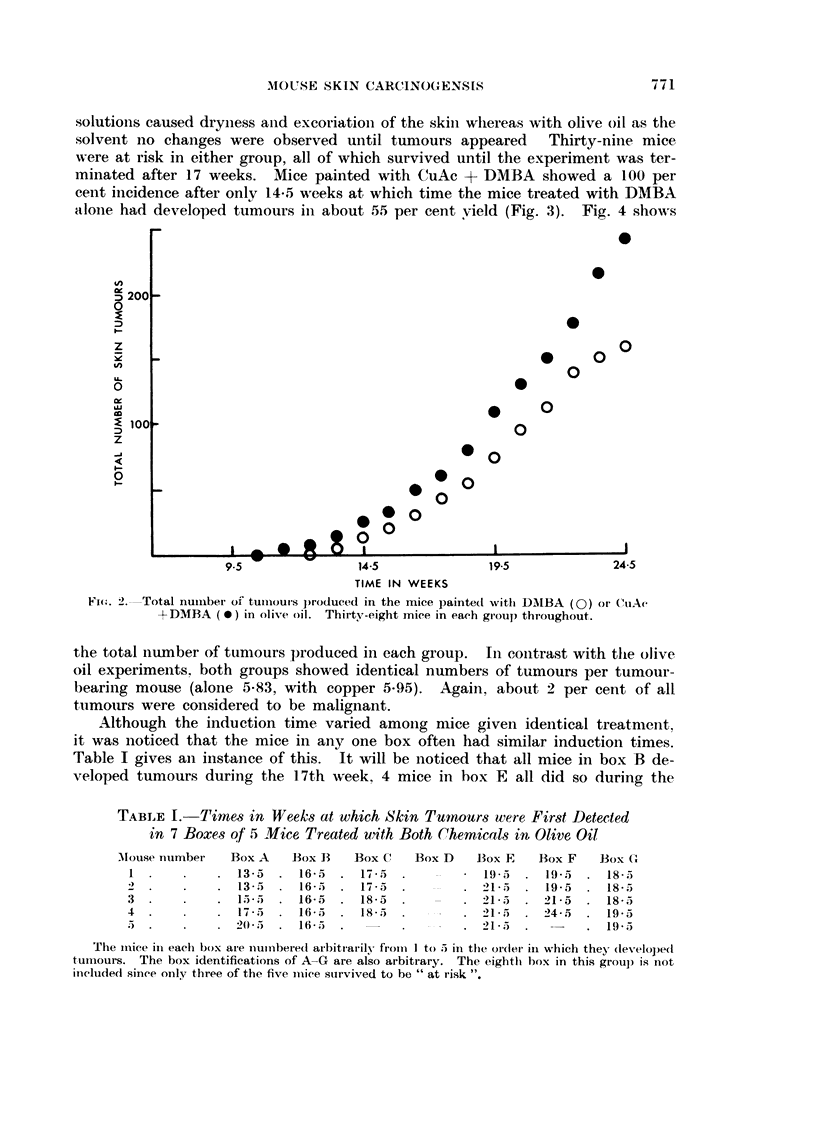

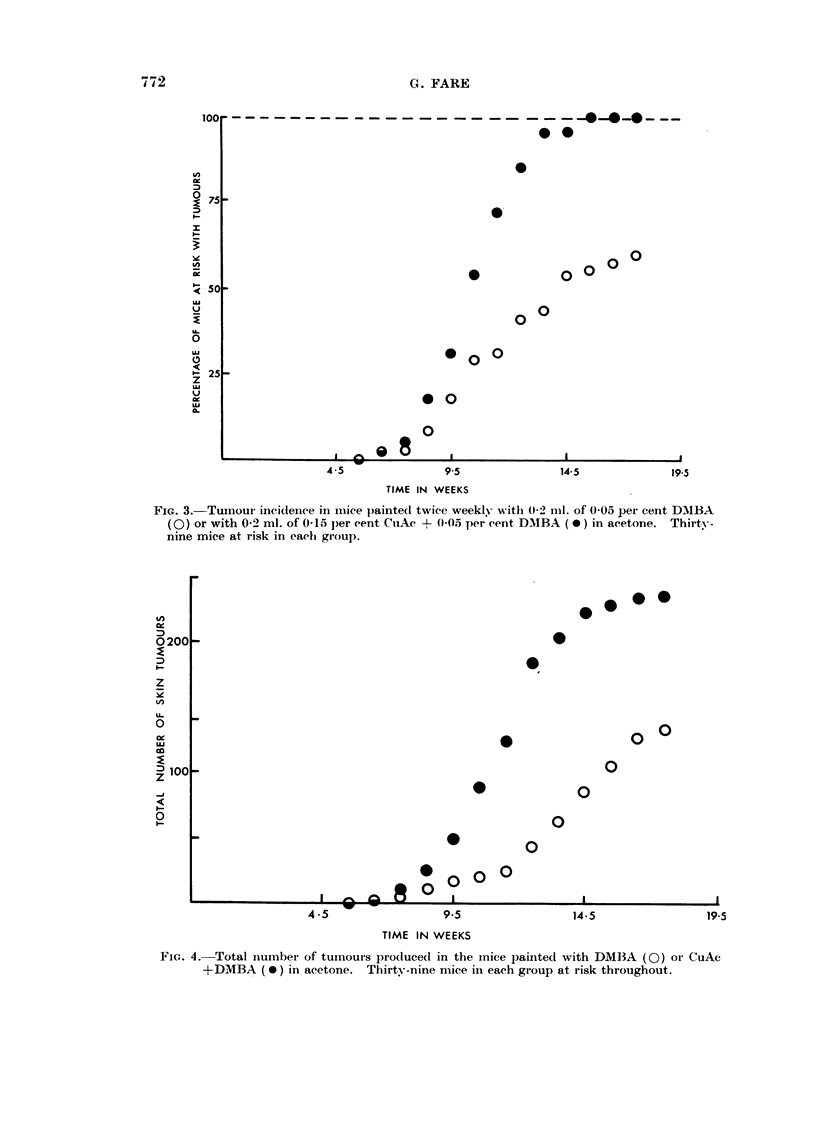

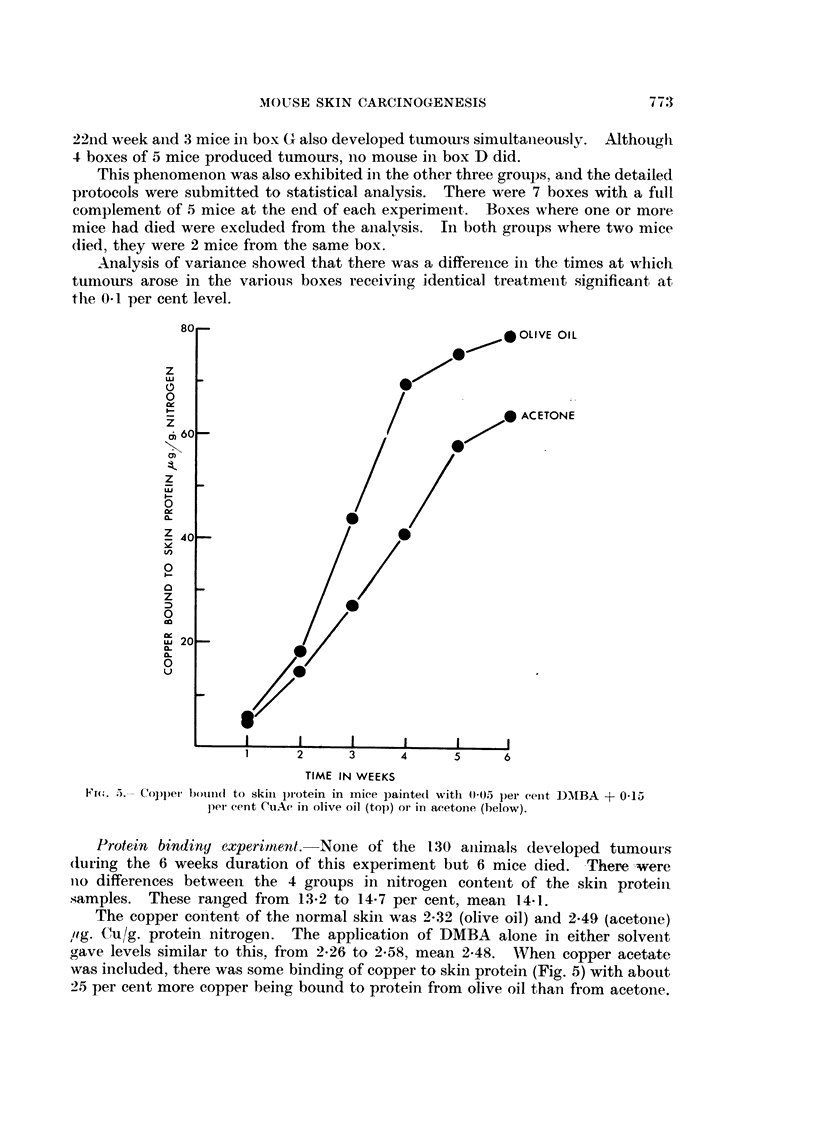

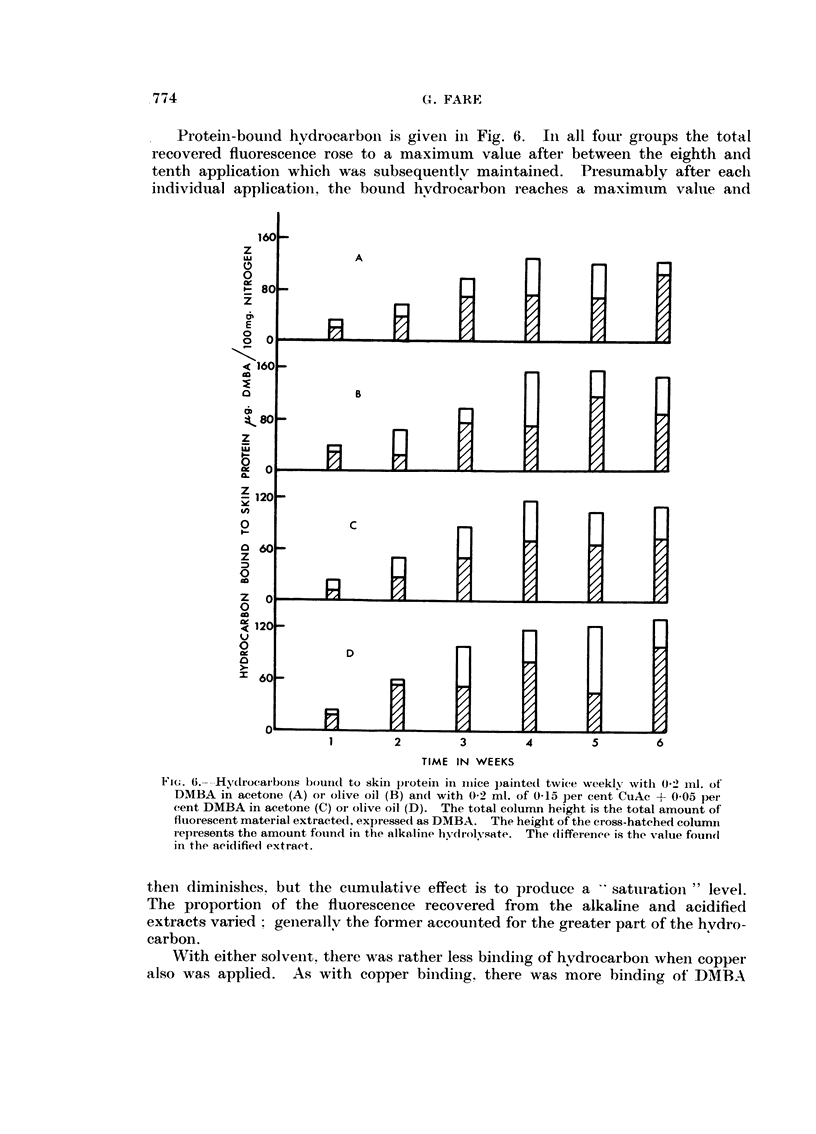

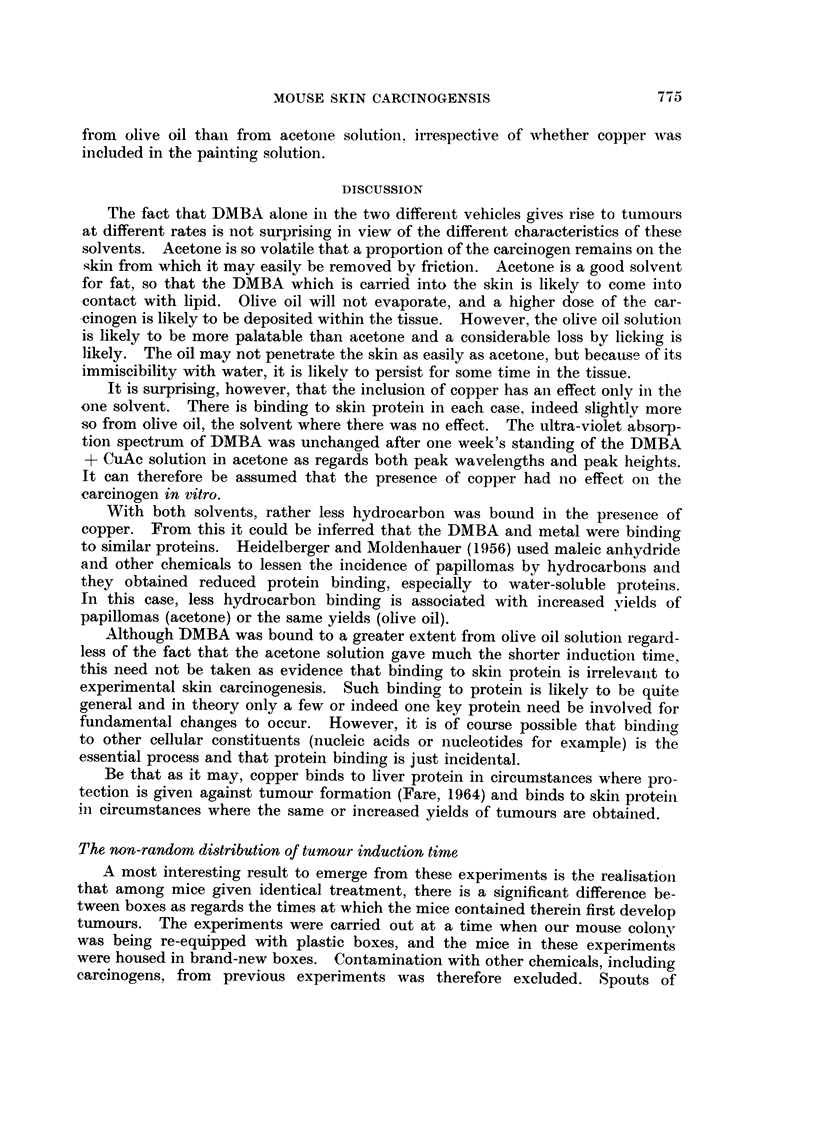

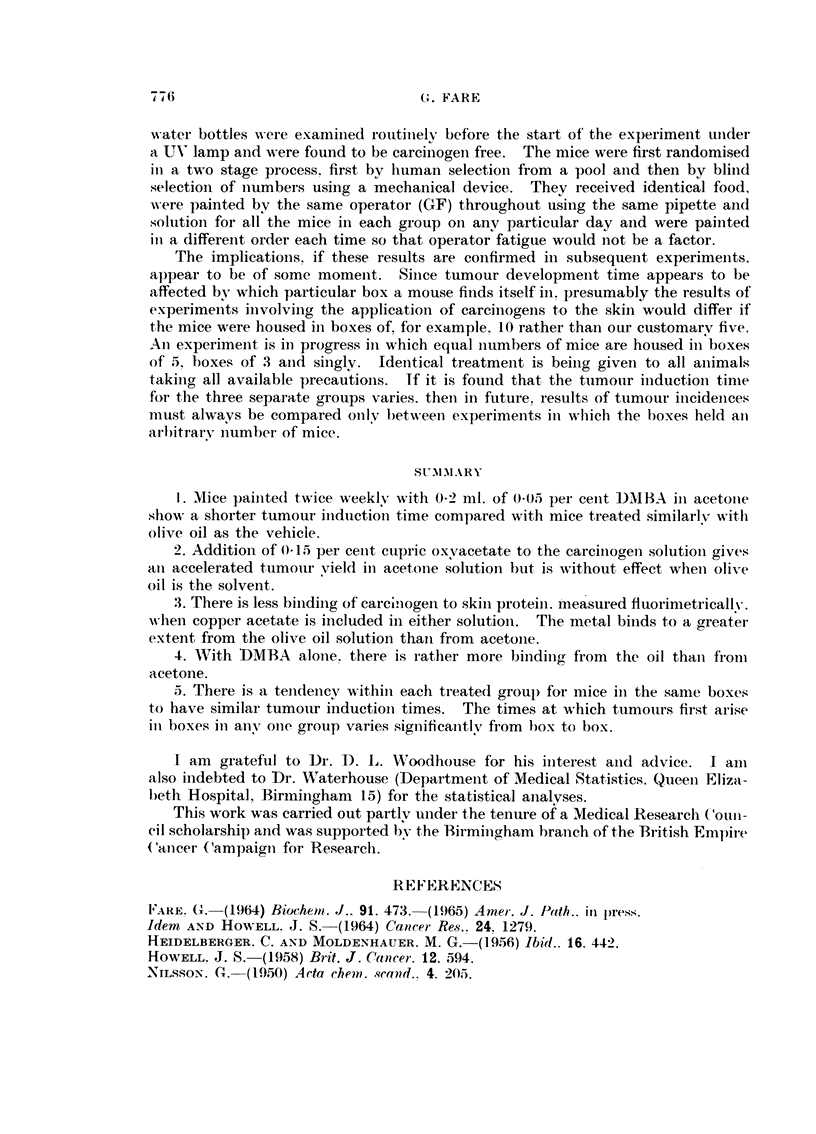


## References

[OCR_00749] HEIDELBERGER C., MOLDENHAUER M. G. (1956). The interaction of carcinogenic hydrocarbons with tissue constituents. IV. A quantitative study of the binding to skin proteins of several C14-labeled hydrocarbons.. Cancer Res.

[OCR_00750] HOWELL J. S. (1958). The effect of copper acetate on p-dimethylaminoazobenzene carcinogenesis in the rat.. Br J Cancer.

